# Quantitative assessment of the microstructure of the mesorectum with different prognostic statuses by intravoxel incoherent motion diffusion-weighed magnetic resonance imaging

**DOI:** 10.1186/s12876-022-02555-9

**Published:** 2022-11-23

**Authors:** Bao-Lan Lu, Yan Chen, Zi-Qiang Wen, Yi-Yan Liu, Yu-Ru Ma, Yu-Tao Que, Zhi-Wen Zhang, Xue-Han Wu, Shen-Ping Yu

**Affiliations:** Department of Radiology, The First Affiliated Hospital, Sun Yat-sen University, 58 Zhongshan Road 2nd, 510080 Guangzhou, People’s Republic of China

**Keywords:** Rectal cancer, Mesorectum, Magnetic resonance imaging, Diffusion

## Abstract

**Background:**

The mesorectum surrounding the rectum provides an ideal substrate for tumour spread. However, preoperative risk assessment is still an issue. This study aimed to investigate the microstructural features of mesorectum with different prognostic statuses by intravoxel incoherent motion diffusion-weighted imaging (IVIM DWI).

**Methods:**

Patients with pathologically proven rectal adenocarcinoma underwent routine high-resolution rectal magnetic resonance imaging (MRI) and IVIM DWI sequences were acquired. The MRI-detected circumferential resection margin (mrCRM) and extramural vascular invasion (mrEMVI) were evaluated. IVIM parameters of the mesorectum adjacent to (MAT) and distant from (MDT) the tumour were measured and compared between and within the prognostic factor groups.

**Results:**

The positive mrCRM (*p*_*MAT*_ < 0.001; *p*_*MDT*_ = 0.013) and mrEMVI (*p*_*MAT*_ = 0.001; *p*_*MDT*_ < 0.001) groups demonstrated higher D values in the MAT and MDT than the corresponding negative groups. Conversely, the positive mrCRM (*p* = 0.001) and mrEMVI (*p* < 0.001) groups both demonstrated lower *f* values in the MAT. Similarly, in the self-comparison between the MAT and MDT in the above subgroups, D showed a significant difference in all subgroups (*p* < 0.001 for all), and *f* showed a significant difference in the positive mrCRM (*p* = 0.001) and mrEMVI (*p* = 0.002) groups. Moreover, the MAT displayed a higher D* in the positive mrCRM (*p* = 0.014), negative mrCRM (*p* = 0.009) and negative mrEMVI groups (*p* < 0.001).

**Conclusion:**

The microstructure of the mesorectum in patients with rectal cancer with poor prognostic status shows changes based on IVIM parameters. IVIM parameters might be promising imaging biomarkers for risk assessment of tumour spread in mesorectum preoperatively.

## Introduction

Colorectal cancer is one of the most common malignant tumours and has a poor prognosis because of local recurrence and metastasis. In rectal cancer, local recurrence mainly occurs as a result of incomplete resection and the mesorectum surrounding the rectum seems to be an ideal substrate for the spreading of tumours [1]. With the proposal of total mesorectal excision (TME), the local recurrence rate of rectal cancer has decreased in the past decades [2, 3]. However, its radicality is limited to risk of undetected metastasis of the mesorectum which can only be directly observed by pathological analysis after the operation. In contrast to intensive imaging studies on the tumour parenchyma, radiology on the mesorectum has not been investigated thoroughly until recently. Although there has been increasing attention given to the assessment of mesorectal morphology by magnetic resonance imaging (MRI) to evaluate the difficulty of resection, resection margin status and prognosis [4–6], few studies have analyzed the microstructure of the mesorectum, which may have profound implications for detecting microtumours in the surrounding mesorectal fat.

A positive circumferential resection margin (CRM) and extramural vascular invasion (EMVI) are risk factors for a poor prognosis of rectal cancer and are also associated with local recurrence and distant metastasis [7–10]. The status of CRM and EMVI are important factors that need to be assessed before making clinical decisions. Given the superior soft-tissue contrast achieved with MRI, recent studies [11–15] have indicated that MRI-detected CRM (mrCRM) and EMVI (mrEMVI) could be used as reliable reference prognostic factors for developing diagnostic strategies.

Functional MRI can provide a visualization of information beyond detailed morphological features and be used to assess the microstructural features of tissue. Intravoxel incoherent motion diffusion-weighed imaging (IVIM DWI) is a novel functional technology that can be used to quantitatively assess pure molecular diffusion and microcirculation in the capillary network (perfusion) without intravenous contrast agent [16, 17]. It has been previously shown that IVIM DWI produces valuable biomarkers for diffusion and perfusion in the mesorectal tissue adjacent to rectal tumours and consequently can be used for purposes other than histological ascertainability [18], suggesting that IVIM DWI might be able to assess the microstructural features of the mesorectum.

Given that positive CRM and EMVI are risk factors for local recurrence and metastasis, and that IVIM DWI is a promising diagnostic tool in rectal cancer, thus, this study aimed to identify the microstructural features of the mesorectum in patients with rectal cancer in term of different prognostic statuses by IVIM DWI and investigate whether IVIM parameters could be imaging biomarker for stratifying different prognosis features.

## Materials and methods

### Patients

This retrospective study was approved by the institutional review board of the First Affiliated Hospital, Sun Yat-sen University with waiver of informed consent.

A retrospective search of the databases of a tertiary referral institution was performed to retrieve all consecutive patients diagnosed with rectal neoplasms who underwent high-resolution rectal MRI examination in our hospital from January 2015 to December 2016 were recruited. Among them, patients who met the following criteria were excluded: (1) carcinoma of the anal canal or sigmoid; (2) lack of histological results, or histologically proven adenoma, gastrointestinal stromal tumour (GIST), mucinous or composition of mucous adenocarcinoma; (3) recurrent rectal carcinoma; (4) underwent neoadjuvant chemoradiotherapy before MRI examination; (5) lack of IVIM DWI sequences; (6) artifacts producing poor image quality; and (7) insufficient space for placing region of interest (ROI) in the mesorectum.

### MRI protocol

All patients were routinely intramuscularly injected with 20 mg of anisodamine to minimize intestinal peristaltic movement 10 min before the examination. Gadopentetate dimeglumine (0.2 mL/kg body weight) was intravenously injected using a power injector at a rate of 3.0 mL/s followed by a 25 mL saline flush.

The rectal MRI examination (Table 1) was performed using a 3 T MRI scanner (Magnetom Verio; Siemens Healthcare) equipped with a six-channel body-matrix coil. Routine rectal MRI included (1) axial, sagittal, coronal and oblique axial (performed perpendicular to the long axis of the rectum at the level of the tumour) T2-weighted imaging and (2) contrast-enhanced fat-saturated oblique axial and 3D coronal T1-weighted imaging. The IVIM DWI sequence was performed prior to gadolinium injection. A total of 14 b values (0, 5, 10, 20, 30, 40, 60, 80, 100, 150, 200, 400, 600 and 1000 s/mm^2^) were applied using a single-shot spin-echo echo-planar-imaging sequence.

**Table 1:** MRI parameters

Parameters	TR/TE (ms)	Number of slices	FOV (mm^2^)	Voxel size (mm^3^)	Acquisition time (s)
**T**_**2**_**WI**					
Axial	3000/87	25	260 × 260	0.8 × 0.7 × 5.0	176
Sagittal	3000/87	19	180 × 180	0.7 × 0.6 × 3.0	150
Coronal	4000/77	24	220 × 220	0.7 × 0.6 × 3.0	172
Oblique axial	3000/84	24	180 × 180	0.7 × 0.6 × 3.0	198
**Post contrast T**_**1**_**WI**					
Oblique axial	716/12	18	180 × 180	0.6 × 0.6 × 3.0	198
Coronal 3D T_1_WI	10/4.9	144	380 × 380	0.8 × 0.8 × 1.6	336
**IVIM DWI**	3800/74.7	21	300 × 245	2.7 × 2.7 × 6.0	301

*FOV* Field of view, *IVIM DWI* Intravoxel incoherent motion diffusion-weighted imaging, *T*_*1*_*WI* T1-weighted imaging, *T*_*2*_*WI* T2-weighted imaging, *TE* Echo time, *TR* Repetition time, *3D* Three-dimensional

### Image interpretation

A computerized radiologic database was used for patient selection and image interpretation. Two consultant radiologists, with more than 5 years of specialized training in rectal MRI independently reviewed the images. Once image interpretation was completed, any disagreement between the observers was resolved by a third investigator (with 25 years of experience in radiology of gastrointestinal tract) and the majority view was taken as the final consensus.

The mesorectal fascia (MRF) is easily identified on axial T2-weighted images as a thin hypointense line. Based on the relationship between the tumour and the MRF, definitive or potentially positive mrCRM involvement was defined as tumour infiltration, suspicious lymph nodes, extramural vascular invasion or disseminated lesions within 1 mm of the MRF [19].

EMVI refers to the invasion of tumour cells into the small vessels outside the rectal wall. mrEMVI was identified as a serpiginous extension of the tumour signal within the vascular structure (in which a vessel was defined as a tubular structure that contained a signal void on T2-weighted images and was continuous across adjacent slices), including a slightly expanded contour and caliber and an obvious irregular vessel contour or nodular expansion of the involved vessels [20].

### IVIM DWI analysis

The data set was analyzed on the basis of the bi-exponential IVIM DWI model introduced by Le Bihan [21] using the following equation: S_b_/ S_0_ = (1 − f) exp (-b·D) + f·exp [ (-b·(D*)], where S_b_ is the signal intensity at the particular b-value used, S_0_ is the signal intensity at the b-value of 0, f is the fraction of the signal linked to microcirculation, D is the true diffusion coefficient representing molecular diffusion of pure water, and D* represents perfusion-related incoherent microcirculation, and is known as the pseudodiffusion coefficient.

The IVIM DWI data were postprocessed using a software developed in house (MATLAB Version 3.1; Mathworks, Natick, MA, USA), and the parameter maps of IVIM DWI (D, D* and *f* parameter maps) were automatically generated in a voxel-by-voxel manner using all 14 b values. A segmented fitting method was used for a more robust calculation, similar to the implementation in a previous study [22]. D and *f* were first estimated by assuming that the perfusion fraction of the signal could be neglected at a b-value > 200 s/mm^2^; then, D* was calculated by applying the previously calculated D and *f* to the IVIM model.

**Fig. 1:**
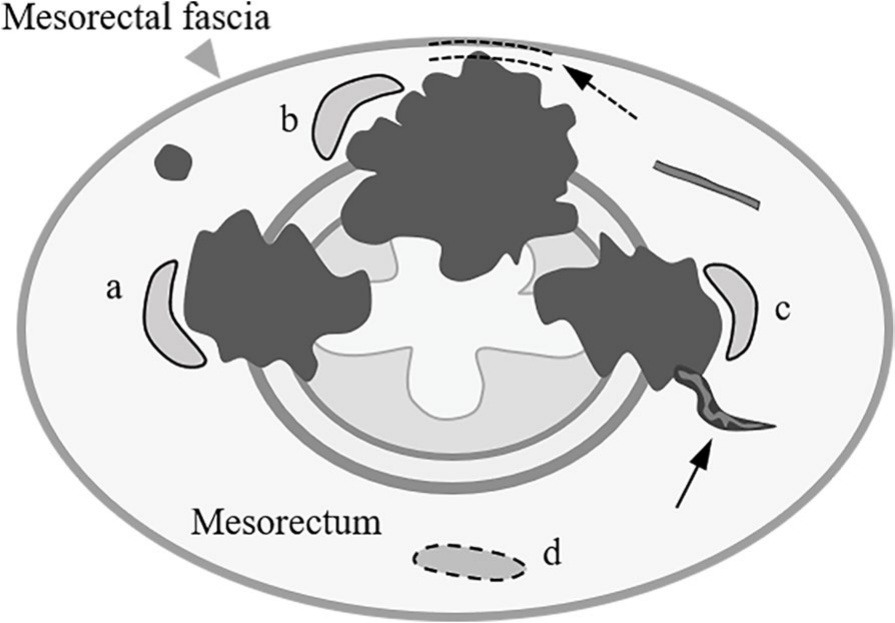
Schematic representation of ROI selection in the mesorectum for different prognostic statuses (dotted arrow indicates positive CRM, solid arrow indicates positive EMVI). The ROIs of the MAT (solid circle a, b and c) were placed within 5 mm of the tumour, or in the upper or lower slice closest to the outline of the largest cross-section of the tumour if there was no available space. The ROIs of MDT (dotted circle d) were placed on the mesorectum contralateral to the semiperimeter tumour cross-section or at least 10 mm away from the full-perimeter tumour cross-section. The ROIs of both the MAT and MDT were drawn to avoid the tumour infiltration area, the invasion vasculature, and suspicious lymph nodes or dissemination

All ROIs were manually delineated and contoured, and they were confirmed by comparing the position in the DWI data (b = 0 s/mm^2^) or D-mapping to that in the axial T2-weighted imaging. The ROIs were placed in the mesorectum adjacent to (MAT, within 5 mm of the outline of the largest cross-section of the tumour or in the upper or lower slices closest to the tumour if there was no available space) and distant from the tumour (MDT, mesorectum contralateral to the semiperimeter tumour cross-section or at least 10 mm from the full-perimeter tumour cross-section) in each case. The sizes of the two ROIs were kept as equal as possible in the same case. Moreover, the ROIs of both the MAT and MDT were drawn to avoid the tumour infiltration area, the invaded vasculature, and suspicious lymph node or dissemination (Fig. 1). The mean value of each IVIM parameter within a ROI on the IVIM parameter maps was recorded for analysis. The mean size of the ROIs of MAT and MDT was 21.12 ± 7.44 mm^2^ and 18.02 ± 7.23 mm^2^, respectively. Additionally, IVIM parameters were processed independently by two radiologists to assess interobserver reproducibility.

### Statistical analysis

Statistical analyse were performed using SPSS v20.0 (IBM Inc., Armonk, NY, USA) and MedCalc Statistical Software v15.8 (MedCalc Software bvba, Ostend, Belgium). All measurements of IVIM parameters were presented using medians (inter-quartile range, IQR). Data distribution normality was assessed using the Kolmogorov–Smirnov or Shapiro–Wilk test. Two independent-sample t or Mann–Whitney *U* tests were used to analyze the differences in the IVIM parameters of the MAT and MDT between the mrCRM-positive/ negative and mrEMVI-positive/ negative groups, respectively. A paired-samples t or Wilcoxon test was used to analyze the differences between the MAT and MDT for each mrCRM and mrEMVI group. The interobserver agreement was assessed with Cohen’s kappa (κ) for evaluating mrCRM and mrEMVI status. κ values of 0–0.20 are characterized as poor agreement, 0.21–0.40 as fair, 0.41–0.60 as moderate, 0.61–0.80 as good, and 0.81–1.00 as excellent. The intraclass correlation coefficient (ICC) was used to assess the interobserver agreement for the IVIM parameters. ICCs > 0.75, 0.5–0.75, and < 0.5 were considered good, moderate and poor agreement, respectively. Moreover, Bland–Altman plots were constructed and limits of agreement (LoAs) based on the plots were estimated to evaluate IVIM measurement reproducibility. A two-tailed *p* value < 0.05 indicated statistical significance.

## Results

### Patient selection and demographics

**Fig. 2:**
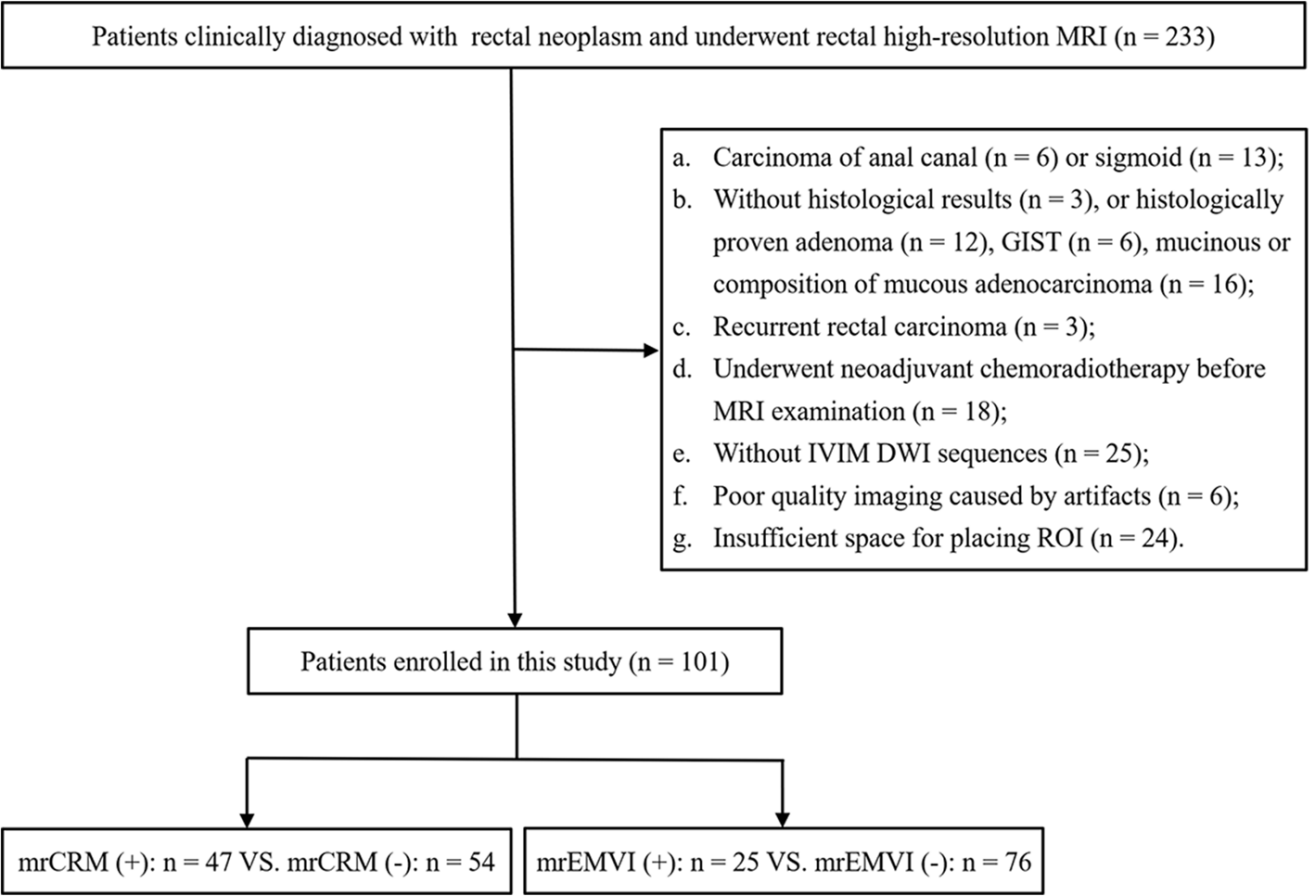
Flow diagram of the study patients. GIST, gastrointestinal stromal tumour; IVIM DWI, intravoxel incoherent motion diffusion-weighted imaging; mrCRM*,* MRI-detected circumferential resection margin; mrEMVI*,* MRI-detected extramural vascular invasion; *ROI,* region of interest

A total of 101 patients were recruited for our study. Forty-seven patients were classified as mrCRM-positive and 54 as mrCRM-negative; 25 patients were grouped as mrEMVI-positive and 76 as mrEMVI-negative (Fig. 2). The patient characteristics are shown in Table 2.

**Table 2:** Patients’ characteristics

**Characteristics**		**Values**
Number		*n* = 101
Age (years)^a^		61 (14)
Sex	Female	38 (37.6%)
	Male	63 (62.4%)
Pathologic T-stage	pT1	2 (2.0%)
	pT2	17 (16.8%)
	pT3	20 (19.8%)
	pT4	31 (30.7%)
	pTx^b^	31 (30.7%)
Pathologic N-stage	pN0	37 (36.6%)
	pN1	22 (21.8%)
	pN2	11 (10.9%)
	pNx^b^	31 (30.7%)
mrCRM	positive	47 (46.5%)
	negative	54 (53.5%)
mrEMVI	positive	25 (24.8%)
	negative	76 (75.2%)

*mrCRM* MRI-detected circumferential resection margin, *mrEMVI* MRI-detected extramural vascular invasion

^a^ Age is expressed as median (IQR)

^b^ nonoperated patients

### Comparison of IVIM parameters between different prognostic status groups in the MAT and MDT

Compared with the mrCRM-negative group, the mrCRM-positive group had a significantly higher D (*p* < 0.001), but a lower *f* (*p* = 0.001) in the MAT. However, only D was significantly different between the two groups in MDT (*p* = 0.013).

Compared with the mrEMVI-negative group, the mrEMVI-positive group had a significantly higher D in both the MAT (*p* = 0.001) and MDT (*p* < 0.001). What’s more, the mrEMVI-positive group demonstrated a significantly lower *f* in both the MAT (*p* < 0.001) and MDT (*p* = 0.003).

**Fig. 3:**
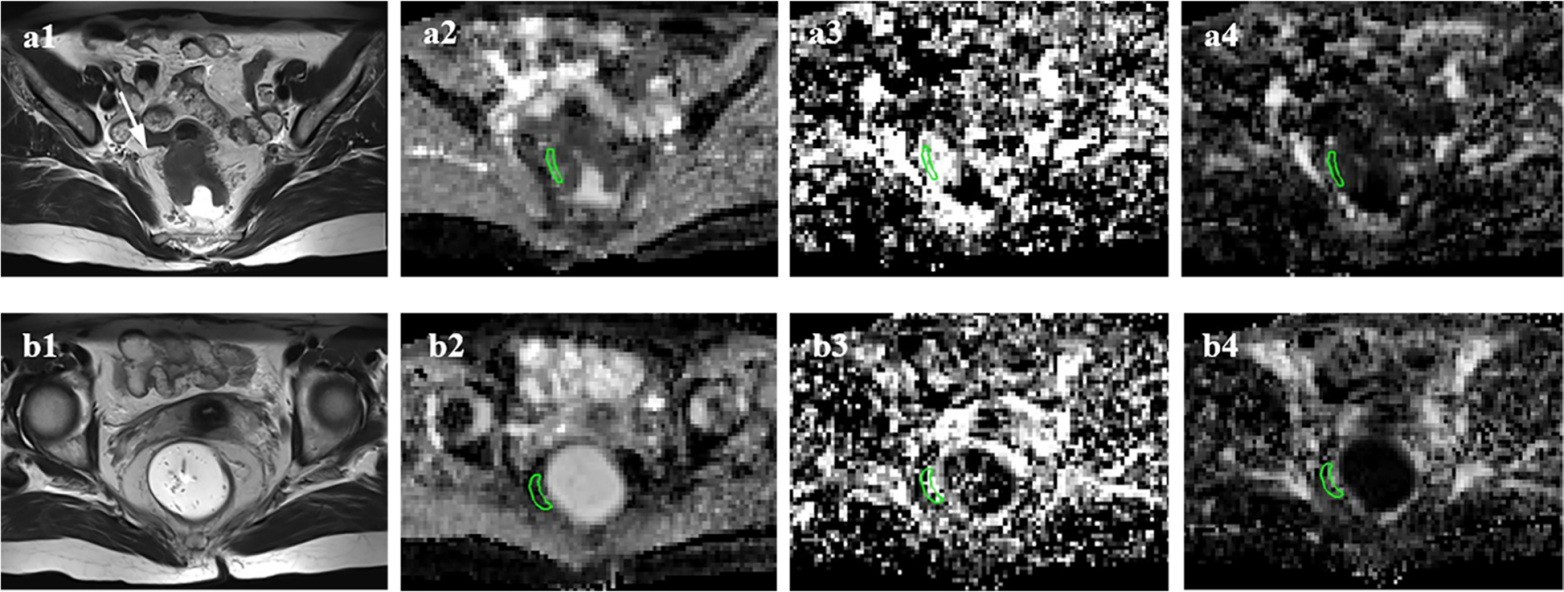
Measurement of IVIM parameters in the mesorectum in patient with positive mrCRM. Axial T2-weighted imaging shows a tumour lying within 1 mm of the mesorectal fascia (**a1**, arrow). The ROIs were delineated in the D (**a2**), D* (**a3**) and *f* (**a4**) maps in the MAT. And the values of D, D* and *f* were 1.38 × 10^–3^ mm^2^/s, 22.95 × 10^–3^ mm^2^/s and 0.21, respectively. The ROIs in the MDT were more than 10 mm away from the largest tumour cross-section (**b1**). The corresponding IVIM maps were showed in **b2**, **b3** and **b4**, and the values of D, D* and *f* were 0.54 × 10^–3^ mm^2^/s, 11.47 × 10^–3^ mm^2^/s and 0.29, respectively

**Fig. 4:**
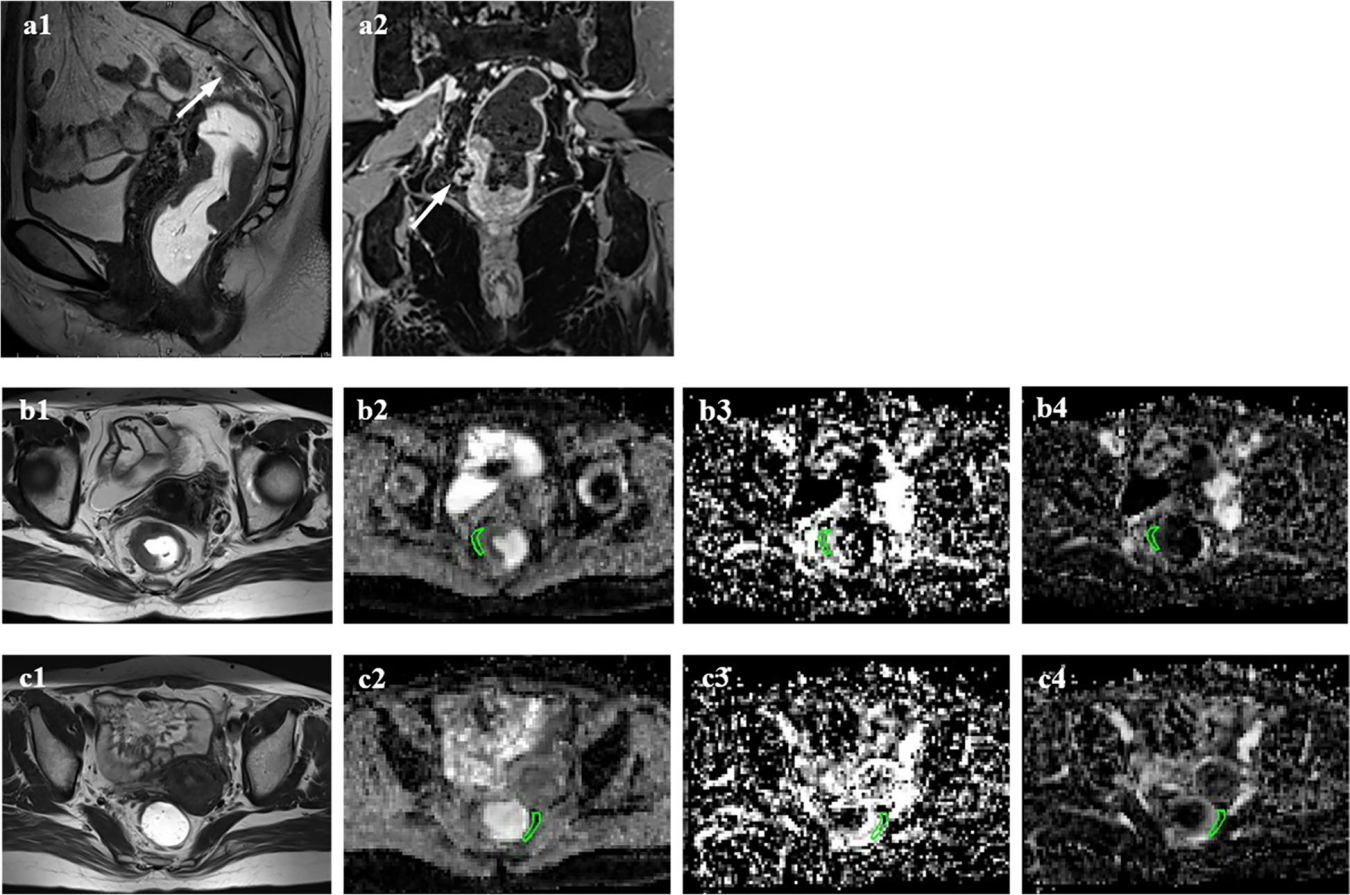
Measurement of IVIM parameters in the mesorectum in patients with positive mrEMVI. Sagittal T2-weighted imaging (**a1**) shows a tubular structure that contains a signal void (arrow). On coronal contrast-enhanced T1-weigheted imaging (**a2**), a small serpiginous vessel outside the tumour demonstrates an irregularly expanded contour (arrow). The ROIs in the MAT (**b2 – b4**) are located within 5 mm of the outline of the largest tumour segment, and the calculated IVIM parameters included D (1.45 × 10^–3^ mm^2^/s), D* (10.79 × 10^–3^ mm^2^/s), and *f* (0.15). The ROIs in the MDT (**c2 – c4**) are located contralateral to the tumour parenchyma as far as possible; D = 0.67 × 10^–3^ mm^2^/s, D* = 12.25 × 10^–3^ mm.^2^/s, and *f* = 0.34

Further details are shown in Table 3. Figures 3 and 4 illustrate examples of measurements of IVIM parameters in the mesorectum in mrCRM-positive and mrEVMI-positive patients, respectively.

**Table 3:** Comparison of IVIM parameters between different mrCRM and mrEMVI statuses in mesorectum adjacent to and distant from the rectal tumours

	**IVIM parameters**	**mrCRM ( +) (*n* = 47)**	**mrCRM (-) (*n* = 54)**	***p***	**mrEMVI ( +) (*n* = 25)**	**mrEMVI (-) (*n* = 76)**	***p***
**MAT**	D (10^–3^·mm^2^/s)	1.31 (0.64)	0.97 (0.47)	< 0.001	1.32 (0.50)	1.00 (0.58)	0.001
	D^*^ (10^–3^·mm^2^/s)	13.43 (6.86)	14.53 (6.08)	0.775	14.35 (7.22)	14.39 (6.27)	0.427
	*f*	0.21 (0.14)	0.29 (0.11)	0.001	0.19 (0.13)	0.29 (0.13)	< 0.001
**MDT**	D (10^–3^·mm^2^/s)	0.73 (0.75)	0.53 (0.31)	0.013	0.88 (0.64)	0.53 (0.33)	< 0.001
	D^*^ (10^–3^·mm^2^/s)	11.98 (5.44)	12.22 (4.95)	0.591	11.36 (7.11)	12.57 (5.06)	0.366
	*f*	0.29 (0.12)	0.31 (0.09)	0.219	0.27 (0.08)	0.32 (0.10)	0.003

*IVIM* Intravoxel incoherent motion, *MAT* Mesorectum adjacent to the tumour, *MDT* Mesorectum distant from the tumour, *mrCRM* MRI-detected circumferential resection margin, *mrEMVI* MRI-detected extramural vascular invasion

### Comparison of IVIM parameters between the MAT and MDT in each subgroup

For the mrCRM-positive group, both D (*p* < 0.001) and D* (*p* = 0.014) were statistically higher in the MAT than in the MDT. Moreover, the MAT had a lower *f* (*p* < 0.001).

For the mrCRM-negative, both the D (*p* < 0.001) and D* (*p* = 0.009) values of the MAT were significantly higher than those of the MDT. However, there was no significant difference in *f* between the MAT and MDT (*p* = 0.325).

For the mrEMVI-positive group, the MAT demonstrated a significantly higher D (*p* < 0.001), but a significantly lower *f* (*p* = 0.002) than the MDT. No significant differences were observed in D* between the MAT and MDT (*p* = 0.176).

For the mrEMVI-negative group, D and D* in the MAT were higher than those in the MDT (*p* < 0.001 for both). However, there was no significant difference in *f* between the MAT and MDT (*p* = 0.066).

### Interobserver agreement evaluation of mrCRM, mrEMVI and IVIM parameters

Good agreement was achieved in the evaluation of mrCRM with a κ of 0.763 (95% confidence interval [CI]: 0.651–0.875; *p* < 0.001). In the evaluation of mrEMVI, the κ value was 0.548 (95% CI: 0.377–0.718; *p* < 0.001), indicating moderate interobserver agreement.

The ICC values for *f* of the MAT and D of the MDT were 0.758 (95% CI: 0.662–0.831, *p* < 0.001) and 0.837 (95% CI: 0.767–0.887, *p* < 0.001), respectively, which both indicated good interobserver agreement. However, the interobserver agreement of the other IVIM parameters of the MAT and MDT were moderate with ICCs ranged from 0.644 to 0.726 (Table 4).

**Table 4:** Intra-class correlation coefficient of IVIM parameters measured by the two radiologists

	**IVIM parameters**	**ICC (95% CI)**	***p***
MAT	D (10^–3^·mm^2^/s)	0.658 (0.531–0.756)	< 0.001
	D^*^ (10^–3^·mm^2^/s)	0.688 (0.570–0.779)	< 0.001
	*f*	0.758 (0.662–0.831)	< 0.001
MDT	D (10^–3^·mm^2^/s)	0.837 (0.767–0.887)	< 0.001
	D^*^ (10^–3^·mm^2^/s)	0.726 (0.619–0.807)	< 0.001
	*f*	0.644 (0.514–0.746)	< 0.001

*CI* Confidence interval, *MAT* Mesorectum adjacent to the tumour, *MDT* Mesorectum distant from the tumour, *ICC* Intra-class correlation coefficient, *IVIM* Intravoxel incoherent motion

**Fig. 5:**
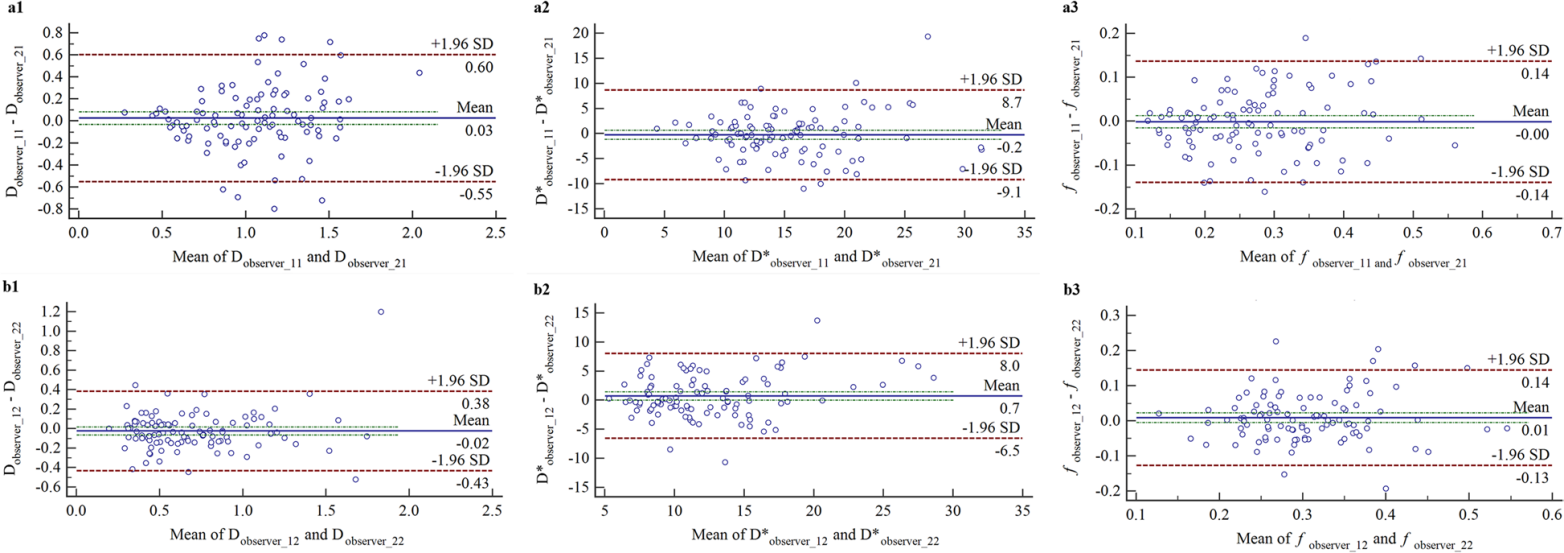
Bland–Altman plots for interobserver agreement of the IVIM parameter measurements in the mesorectum. For difference, the 95% LoAs is -0.55 to 0.60 for D (a1), -9.1 to 8.7 for D* (a2), -0.14 to 0.14 for *f* (a3) in MAT, and -0.43 to 0.38 for D (b1), -6.5 to 8.0 for D* (b2), -0.13 to 0.14 (b3) for *f* in MDT

Moreover, except for D* of the MDT (*p* = 0.046), the Bland–Altman plots showed that the differences in the other IVIM parameters of the MAT and MDT (all *p* > 0.05) did not deviate significantly from the equality line (difference = 0), indicating good agreement between the two radiologists. The 95% LoAs of the differences are shown in Fig. 5.

## Discussion

This study investigated the microstructural features of the mesorectum in patients with rectal cancer with poor prognostic factors, including positive mrCRM and mrEMVI, by using IVIM DWI. Our results showed that the IVIM parameters of the MAT with poor prognostic factors were significantly different (higher D and lower *f)*, implying that the microstructural status of the mesorectum changes in some respects. IVIM parameters D and *f* might be promising imaging biomarkers for stratifying different prognosis groups.

The mesorectum surrounding rectal tumours is as important as the tumour itself, as it is rich in lymphatic and vascular tissue, which provides an ideal substrate for tumour spread. A study indicated that both macrovascular and microvascular function in mesorectum surrounding rectal tumours displayed several abnormal features, including high vascular branching, an enlarged vascular lumen and enhanced microvascular function, relative to the mesorectum surrounding the normal rectal wall. Moreover, the formation of highly vascular stroma precedes actual tumour invasion [23]. Angiogenesis is the basis of tumour growth and metastasis. Hence, we hypothesized that the microvasculature of the mesorectum near the tumour may be increased.

Regarding different prognostic statuses, in our study, the perfusion related coefficient *f* of the MAT showed a lower value in the positive mrCRM and mrEMVI groups than in the corresponding negative groups. Inconsistency, previous studies have indicated that the mesorectum surrounding tumours had increased blood flow and enhanced microvascular function on dynamic contrast-enhanced MRI [23, 24]. Nevertheless, *f* is a reflection of effective perfusion, and angiogenesis in the tumour infiltration area may be irregular hyperplasia vessels that cannot provide effective perfusion [17, 25]. Thus, the functional status of tumour angiogenesis was disrupted in the MAT groups, resulting in a decrease in *f* in the mrCRM- and mrEMVI-positive groups. A similar result was also observed in comparing *f* in the MDT between the positive and negative mrEMVI groups. It may be that the mrEMVI-positive patients had more aggressive tumour invasion that involved the microstructure of the MDT.

Unexpectedly, there was no significant difference in the perfusion correlation coefficient D* of the MAT and MDT in patients with different prognostic statuses. However, a previous study showed that the patients with EMVI had a lower D* than patients with no EMVI in the assessment of the tumour parenchyma [26]. Further studies are warranted to explore the implementation of D* in the assessment of the mesorectum.

IVIM DWI can provide information of perfusion and diffusion simultaneously without contrast agent administration. D is a coefficient reflecting the extent of restricted pure molecule diffusion. Interestingly, D increased with a more aggressive status in the positive mrCRM and mrEMVI groups in our study. This result was probably due to the basic characteristics of the mesorectum. Our result is supported by a previous study in which D was lower in mesorectal fat than in the rectum and tumour [18]. The normal mesorectum consists of large fat cells and a small intercellular space that may lead to remarkable a restriction in molecular diffusion that counters the influence of tumour invasion. In further assessments based on the mrCRM and mrEMVI subgroups, similar results were also observed in the comparison between the MAT and MDT.

For the evaluation between the MAT and MDT in each subgroup, importantly, our study showed that the mrCRM- and mrEMVI-positive groups both demonstrated a decreased *f* in the MAT. However, there was no difference between the MAT and MDT in either mrCRM- or mrEMVI-negative group. This is inconsistent with the results of a previous study, which showed significantly enhanced microvascular function in the tumour surrounding mesorectum relative to the normal mesorectum [23]. The authors attributed this similarity to the fact that the functional status and the anatomic structure of the tumour vasculature do not always coincide, resulting in a decreased effective perfusion fraction [25]. Furthermore, we performed subgroup analyses, whose results may explain the difference in results between the positive and negative subgroups in this study. Additionally, our presented method was different from dynamic contrast-enhanced MRI which was used in the previous study.

Although our study revealed a greater D* for the positive mrCRM, negative mrCRM and mrEMVI subgroups in the MAT than in the MDT, no significant difference was observed in the positive mrEMVI subgroup. This result should be carefully interpreted because the sample of mrEMVI- positive patients was small. It is likely that further studies will be needed to explore the implementation of D* derived from IVIM DWI to evaluate the microstructure of the mesorectum.

There are several limitations in our study. First, the involvement of CRM and EMVI was not confirmed by surgery. Nevertheless, the mrCRM and mrEMVI assessments based on high-resolution MRI are accurate, as previous studies [11–15] have reported, and we accepted the final consensus of a third senior investigator when there was disagreement between the observers. Second, the influence of the thickness or volume of the mesorectum was not taken into account. The small size of the mesorectum may limit the placement of ROIs, especially for ROI selection in the MDT. We did not exclude the full-perimeter lesions, which may also have some influence in selecting ROIs in the MDT. To minimize the bias resulting from the selection of ROIs in the MDT, we excluded cases without sufficient space to draw the ROIs. Third, T and nodal stage were not included as prognostic factors in this study. It is feasible that the involvement of MRF may also play a role in the tumour infiltration. Whilst including T3 substages will be more worthy of further study [27, 28]. For nodal stage, lymph node involvement determined by imaging still remains challenging with well-known inaccuracies and is less apparent [29, 30].

## Conclusions

This study demonstrated that the IVIM parameters of mesorectum with poor prognostic factors were significantly different in some respects, which may indicate microstructural changes in the mesorectum. IVIM DWI is a feasible technique for reflecting the status of the mesorectum, and the parameters D and *f* might be a potential promising imaging biomarkers for stratifying different prognosis groups and predicting a high risk of undetected metastasis in the mesorectum.

## Data Availability

The data that support the findings of this study are available on request from the corresponding author (Shen-ping Yu). The data are not publicly available because the aforementioned data contain information that could compromise the privacy of the research participants.

## Abbreviations

CRM: Circumferential resection margin
EMVI: Extramural vascular invasion
FOV: Field of view
GIST: Gastrointestinal stromal tumour
ICC: Intraclass correlation coefficient
IVIM DWI: Intravoxel incoherent motion diffusion-weighted imaging
IQR: Inter-quartile range
LoAs: Limits of agreement
MAT: Mesorectum adjacent to the tumour
MDT: Mesorectum distant from the tumour
mrCRM: The MRI-detected circumferential resection margin
mrEMVI: The MRI-detected extramural vascular invasion
MRF: Mesorectal fascia
MRI: Magnetic resonance imaging
ROI: Region of interest
TE: Echo time
TME: Total mesorectal excision
TR: Repetition time
3D: Three-dimensional
